# Effect of New Frying Technology on Starchy Food Quality

**DOI:** 10.3390/foods10081852

**Published:** 2021-08-11

**Authors:** Yi Wang, Xianglei Wu, David Julian McClements, Long Chen, Ming Miao, Zhengyu Jin

**Affiliations:** 1 School of Food Science and Technology, Jiangnan University, 1800 Lihu Road, Wuxi 214122, China; 6190111083@stu.jiangnan.edu.cn (Y.W.); 1012190424@stu.jiangnan.edu.cn (X.W.); fpcenter@jiangnan.edu.cn (Z.J.); 2 State Key Laboratory of Food Science and Technology, Jiangnan University, 1800 Lihu Road, Wuxi 214122, China; miaoming@jiangnan.edu.cn; 3 Department of Food Science, University of Massachusetts, Amherst, MA 01003, USA; mcclements@foodsci.umass.edu

**Keywords:** vacuum frying, microwave vacuum frying, air frying, food quality

## Abstract

Frying is commonly used by consumers, restaurants, and industries around the globe to cook and process foods. Compared to other food processing methods, frying has several potential advantages, including reduced processing times and the creation of foods with desirable sensory attributes. Frying is often used to prepare starchy foods. After ingestion, the starch and fat in these foods are hydrolyzed by enzymes in the human digestive tract, thereby providing an important source of energy (glucose and fatty acids) for the human body. Conversely, overconsumption of fried starchy foods can promote overweight, obesity, and other chronic diseases. Moreover, frying can generate toxic reaction products that can damage people’s health. Consequently, there is interest in developing alternative frying technologies that reduce the levels of nutritionally undesirable components in fried foods, such as vacuum, microwave, air, and radiant frying methods. In this review, we focus on the principles and applications of these innovative frying technologies, and highlight their potential advantages and shortcomings. Further development of these technologies should lead to the creation of healthier fried foods that can help combat the rise in diet-related chronic diseases.

## 1. Introduction

Deep frying is a widely used cooking process, which has been applied to prepare various kinds of foods with unique textures, flavors, and appearances since ancient times [1]. Frying typically involves cooking foods at relatively high temperatures for short times using a liquid oil as a heating medium [2]. During frying, mass and heat transfer processes occur simultaneously: heat and oil move into the food, while moisture moves out [3]. The high frying temperatures used, typically mean than only short times (0.5 to 5 min) are required to fully cook foods [4]. Moreover, frying can kill microorganisms in food more effectively than many other cooking methods, and the high fat and low water content of the fried food produced is not conducive to the growth and reproduction of microorganisms. The absorption of oil during frying increases its calorie content, which may be undesirable from a nutritional perspective [5]. Moreover, the relatively high temperatures involved during frying can lead to the chemical degradation of some heat-labile food components.

Starch is an important source of energy in the human diet. It also plays an important role as a functional ingredient in the food and pharmaceutical industries [6,7,8]. Cereal starch currently accounts for more than 80% of the total global starch market [9] and it is often used in the production of fried starchy foods. Starch is the main component in many fried foods, such as potato chips, French fries, instant noodles, and coating flours. During frying, the oil content increases by an amount that depends on the initial composition and structure of the food product. A large amount of oil is absorbed by starch granules, with most of it being located near their surfaces [10]. The structure and physicochemical properties of starch are affected by the heat and mass transfer processes during frying, thus changing the final quality of starch-based foods [11]. However, little information is currently available on the changes in properties of starch that occur when it is heated in oils at the high temperatures used during frying [12].

A growing number of people are concerned about food- and nutrition-related issues due to the increasing prevalence of diet-related chronic diseases in many countries. Foods typically absorb a large amount of oil during frying, leading to around 30 to 50% fat in the final product [13]. Excessive fat intake, especially the saturated- and trans-fats often used during industrial frying to avoid oxidation, leads to obesity and some chronic diseases, such as cardiovascular and cerebrovascular diseases, and several types of cancers [14]. Besides, the high temperatures used during frying can generate potentially harmful substances. For example, fried foods containing starch and protein, such as French fries and potato chips, are the main source of acrylamide in many people’s diet, which may be a human carcinogen [15]. This toxic substance is generated as the result of the Maillard reaction between amino acids (mainly asparagine) and reducing sugars (mainly glucose) at high temperatures [16]. The concentration of acrylamide formed depends on several factors, including food type, moisture content, and frying conditions. Moreover, the type of frying oil, especially the composition, is very important for the final quality and safety of fried foods. Fatty acid composition, and advantages and disadvantages of different edible oils are shown in Table 1. Unsaturated fats are susceptible to lipid oxidation during frying, which can also lead to the generation of toxic reaction products. Extra Virgin Olive Oil (EVOO) is mainly composed of triglycerides, with relatively high concentration of oleic acid, monounsaturated fatty acid and low polyunsaturated fatty acid amounts, which makes it more stable at higher temperatures than other edible oils with high polyunsaturated fatty acid amounts [17,18]. In other words, EVOO is more stable during oxidation and heating than other edible oils [19]. According to the results obtained by Napolitano et al. [20], the more polyphenols in olive oil used for fried potatoes, the less acrylamide is formed. This is in agreement with the study of Friedman and Levin [21], who reported that antioxidants can reduce the level of acrylamide in heated foods. Although soybean oil contains high levels of polyunsaturated fatty acids, and is therefore highly susceptible to oxidation, it is still widely used in starch-based fried foods due to its low price [22]. Liu et al. [23] speculates that fried foods may absorb harmful compounds from frying oils during the cooking process, especially when foods are fried for prolonged periods. The type of oil also affects the formation of acrylamide. According to the results reported by Gertz and Klostermann [24], when French fries are fried in palm oil, levels of acrylamide are higher in French fries than in rapeseed oil. Becalski [25] revealed that the acrylamide content of the sample fried with olive oil is higher than that of the sample fried with corn oil.

Due to concerns about the potentially negative health effects of frying, both pre-frying and post-frying techniques have been developed to reduce the oil absorption of fried samples [26,27]. Other studies have shown that the content of amylose in starch samples also has an effect on the oil absorption of starch fried foods, and the higher the content of amylose, the lower the oil absorption of potatoes [28]. The efficacy of a number of innovative frying technologies have been investigated. These technologies may be able to produce fried foods with lower total oil content and reduced levels of potentially toxic substances, while still maintaining desirable quality attributes, thereby leading to a new generation of healthier and delicious fried foods. 

To achieve this goal, researchers have been carrying out research to improve the effectiveness of a variety of frying technologies that were developed recently or in the past [29]. For example, vacuum frying, which originated around 60 years ago, can be used to fry foods in the absence of air [30]. Some researchers have combined microwave and ultrasonic methods with vacuum frying to further improve the quality of the fried foods produced [31]. In addition, other technologies that use different heat-transfer mediums or heating sources to fry foods have also been investigated, including air frying [32], spray frying [33], radiant frying [34], oil–water mixed frying [35], and electric field frying [36]. Different frying methods lead to different product characteristics and have different suitabilities for large-scale industrial production of fried products. The overall characteristics and applications of some novel frying techniques are summarized in Table 2. In this paper, we review several of the most promising new frying technologies available, focusing on the application to cooking starchy foods. In particular, we present their operating principles and discuss their effects on product quality and safety. Moreover, we discuss their current limitations and their potential for commercial development. In future, it is likely that one or more of these technologies may at least partially replace traditional frying methods and produce better-quality foods in the future.

## 2. Vacuum Frying

Deep-frying is a high-temperature short-time heating process that is widely used to prepare starchy foods. The foods produced using this method have a golden color, crispy texture, and unique flavor profile, which is extremely popular with many people. Vacuum frying is a new food processing technology developed in the late 1960s and early 1970s that is carried out under pressures well below atmospheric levels. Compared with conventional frying, vacuum frying has several advantages: (1) the oil absorption rate is decreased [48]; (2) the formation of harmful substances (such as acrylamide) during frying is reduced due to the lower temperatures and pressures used [49]; (3) the nutritional quality of the food is better preserved [50]. For these reasons, vacuum frying is often used in the dehydration and drying of fruits and vegetables [51]. Vacuum frying has a wide range of applicability, which is mainly used in fruits (e.g., apples, bananas, strawberries), vegetables (e.g., carrots, tomatoes, potatoes, onions), and dried fruits and nuts (e.g., dates, peanuts).

### 2.1. The Operating Principles of Vacuum Frying

According to the frying pressure used, deep frying can be divided into three categories: conventional, vacuum, and high-pressure frying. Compared with conventional frying, the food quality produced by vacuum frying is higher. The boiling point of water is around 100 °C under atmospheric pressure, but is considerably lower in a vacuum, which means that lower temperatures can be used during vacuum frying than conventional frying. Studies have shown that the internal temperature of a food rises rapidly during vacuum frying until it reaches the boiling point of water (under the relevant pressure), and it then remains at this temperature until the water evaporates, and then increases steadily until it reaches the temperature of the surrounding oil [52]. During frying, water is lost from the food in the form of water vapor, and oil enters, so that vacuum-fried products absorb lower amounts of oil [53].

**Figure 1:**
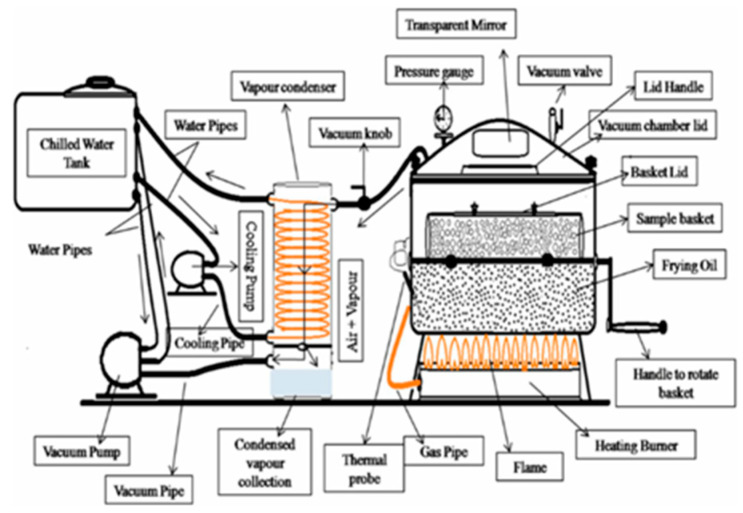
A schematic diagram of the vacuum frying system for industrial production [54].

As the pressure in the system decreases, so does the boiling point of water. Under a vacuum, the water in fried food can reach its boiling point and fully evaporate at a lower temperature. A vacuum frying system typically consists of three units: a vacuum frying chamber, vapor cooling condenser, and high-intensity vacuum pump (Figure 1) [54]. In the vacuum frying system, the vacuum pump is opened before frying to keep the system in a vacuum state. The oil is heated by an electric heater or gas burner under the frying chamber. Before frying, the food is placed in the sample basket. When the oil temperature reaches a predetermined temperature, the basket net is immersed in hot oil through the rotating handle. During the frying process, the food is heated, water is evaporated, and the water vapor released flows into the condensed vapor collection unit through a vacuum pipe.

### 2.2. Effect of Vacuum Frying on Food Safety

In conventional frying, the oil is in a high-temperature state and in contact with air and water. Under these conditions, oil may undergo a series of complex chemical reactions, such as oxidation, polymerization, and hydrolysis, resulting in the production of a variety of harmful substances. For instance, acrylamide, funan, epoxy propionamide, propylene, polycyclic aromatic hydrocarbons, trans-fatty acids, and other harmful substances may be produced during frying. A potential advantage of using vacuum frying is that the production of these toxic substances is reduced because of the lower temperatures employed. Lozano-Castellon et al. [55] compared the changes of olive oil in deep frying and vacuum frying, and the results showed that vacuum frying preserved the phytochemical characteristics of EVOO, and traditional frying produced more oxidation products (such as hydroxy-derivatives of lipids).

Acrylamide (CH_2_=CHC(O)NH_2_) is widely detected in fried foods with high starch content as a result of the Maillard reaction [56]. This substance has been reported to exhibit neurotoxicity, genotoxicity, and carcinogenicity [57]. The hydrolysis of acrylonitrile has been identified as the main path leading to the formation of acrylamide during frying. Williams et al. [58] showed that the acrylamide content of potato chips is mainly controlled by the reducing sugar level rather than the asparagine level. Several factors affect the formation of acrylamide in fried products, including the composition of the raw materials (such as asparagine, glucose, and water contents), as well as the frying conditions used (such as temperature, time, and pH value) [59]. The formation of acrylamide and other potentially toxic reaction products is reduced during vacuum frying because of the lower temperatures used. In addition, the reduced oxygen levels used in vacuum frying inhibit the formation of harmful substances due to oxidation of oils and proteins [60,61]. 

Granda and Moreira [62] found that the content of acrylamide in vacuum-fried potato chips was significantly lower than that in conventional fried ones, which may lead to healthier products. In another study, it was reported that the content of acrylamide in vacuum-fried potato chips decreased by 94% compared to conventionally fried ones [49]. The authors also reported that both the temperature and time of vacuum frying affected the acrylamide content in the final product.

Funan is another harmful substance produced during high temperature processing, which is included in the list of 2B carcinogens by the World Health Organization. Thermal degradation and rearrangement of sugars and amino acids, and thermal oxidation of polyunsaturated fatty acids and ascorbic acids are considered to be the main pathways for funan formation in food products [63]. Like acrylamide, reducing the frying temperature helps to reduce the funan content in the final product [64]. The funan content of vacuum-fried products has been reported to be decreased by 81, 46, and 34% under atmospheric pressures corresponding to frying temperatures of 96, 106, and 116 °C, respectively [65]. Table 3 shows the results of the samples under different frying conditions, highlighting the differences between conventional and vacuum frying under several air pressures. 

### 2.3. Effect of Vacuum Frying on Food Quality

The operating pressure, frying temperature, and frying time used during vacuum frying affect the physical and chemical properties of fried products, such as oil absorption, water loss, color, and texture.

Compared with conventional atmospheric pressure and high temperature frying, the oil content of foods can be greatly reduced by vacuum frying in a closed system [66]. For instance, Garayo and Moreira et al. [67] reported that the microstructure of vacuum-fried potato chips was different from that of conventionally fried ones. In particular, there were differences in the number and size of the stomata on the surfaces of the potato chips, which led to lower oil absorption for the vacuum-fried potato chips. Oil uptake has also been reported to be lower for vacuum-fried than conventional fried pear slices [68] and banana slices [69]. In conventional frying, the increase in the temperature of the product from the ambient temperature to the boiling point of water causes the water in the product to evaporate rapidly. However, during this period, some of the vaporized moisture is trapped within the pores in the plant tissue, which inhibits oil absorption. However, when the fried food is removed from the hot oil, its temperature decreases rapidly, thereby causing the moisture vapor trapped in the pores to condense and the pressure in the pores to decrease. As a result, some of the oil that was originally adsorbed on the surfaces of the fried food is absorbed through the pores of the chip, so as to increase the total amount of oil absorption [70,71]. In vacuum frying, however, because of the lower frying temperatures and pressures used, the samples have fewer open pores, which means that less volatilized moisture is trapped [72]. In addition, the lower frying temperatures used reduce the cooling time of the finished product. As a result, there is less time for oil to enter the voids and replace the water, which is also greatly conducive to the reduction of the overall oil content after frying [73].

Due to the absence of air (oxygen) during vacuum frying, chemical degradative reactions, such as oxidation and enzymatic browning, are inhibited. As a result, the original color and nutrients of the fried products can be preserved to a greater extent [65]. Garayo and Moreira [67] studied the color of vacuum potato chips. They showed that there were significant differences in color (L*, a*, b*) between these products and those produced using conventional frying, with the overall appearance of the vacuum-fried products being superior. According to Tan et al. [74], the total color of donuts by vacuum frying increased with the increase of frying temperature, but was independent of vacuum.

The textural attributes of fried products, such as their brittleness or hardness, are also important for determining their overall quality. The microstructure of fried products changes during cooking, which affects their textural properties. Dueik et al. [75] reported no significant difference in the brittleness of vacuum and atmospheric pressure fried carrot chips. In contrast, Troncoso et al. [76] found that the hardness and brittleness of vacuum-fried potato chips were significantly lower than those of atmospheric pressure-fried ones. However, sensory evaluation showed that the vacuum-fried potato chips had higher scores and better-quality attributes.

## 3. Microwave Vacuum Frying 

As discussed in the previous section, there has been great interest in the production of vacuum-fried products because of their enhanced color, texture, and nutritional attributes. However, researchers have shown that the potential of vacuum frying can be further improved by combining it with other technologies [77]. In particular, there has been interest in the development of microwave vacuum frying (MVF), which uses a microwave oven to heat foods placed in a vacuum [78]. 

During MVF, a microwave is used as a heating source to overcome the shortcomings of traditional vacuum frying [41]. Compared with other frying methods, the main advantage of MVF is that it is a faster dehydration method, which can produce fried fruit and vegetable products with lower oil content, better texture and flavor, less harmful substances, and higher overall quality attributes [79]. These benefits are mainly a result of the reduced heating times required when a microwave is used to heat the samples rather than a normal oven.

### 3.1. The Working Principle of Microwave Vacuum Frying

**Figure 2:**
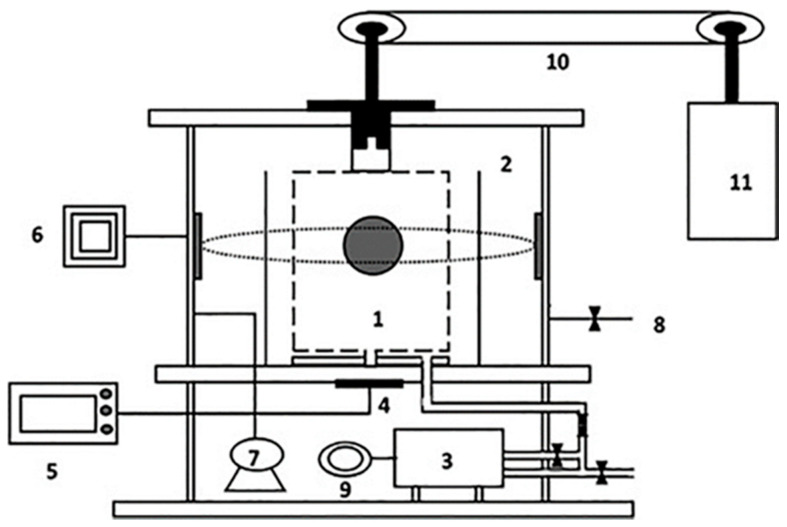
Schematic diagram of the microwave vacuum frying equipment: (1) Frying chamber; (2) vacuum chamber; (3) oil tank; (4) temperature sensor; (5) controller; (6) microwave source; (7) vacuum pump; (8) valve for breaking vacuum; (9) circulation pump; (10) conveyor; and (11) electric motor [84].

Microwaves are a form of electromagnetic radiation with a wavelength range from around 1 mm to 1 m, which corresponds to a frequency range from around 0.3 GHz to 300 GHz. Microwaves are used to heat dielectric materials by inducing molecular vibrations as a result of dipole rotation or ionic polarization [80]. The heating mechanism of MVF is therefore different from that of conventional convection heating. The dipolar rotation mechanism is the primary principle of microwave dielectric heating in foods [81]. During MVF, heat is generated and transferred within the product due to the rapid rotation of polar molecules under rapidly oscillating electric fields [82]. The rapid movement of the polar molecules and ions within the product generates heat, which leads to fairly uniform heating throughout the product [83]. Due to the relatively high internal pressure associated with the production of steam within a food product exposed to microwaves, heat is rapidly transferred, resulting in rapid drying without overheating the product surface [43]. Figure 2 shows the operating principles of each part of a microwave vacuum fryer.

### 3.2. Effect of Microwave Vacuum Frying on Food Safety 

Microwave vacuum frying reduces the boiling point of water in foods, meaning that lower temperatures and shorter times are required for effective dehydration, which decreases the rate of Maillard, lipid oxidation and enzymatic browning reactions. As an example, Sahin et al. [85] reported that when the frying temperature and residual moisture content were similar, the acrylamide content of potato chips fried for 1 min at 400 W microwave power was around 88% lower than that of potato chips fried for 4.5 min in a conventional way.

Sansano et al. [42] reported that the acrylamide content of fried foods decreased with increasing microwave power. However, after reaching a certain frying time, acrylamide formation increased with microwave power. This discovery corresponds with the results of Sahin et al. [85], because with the increase of microwave power and frying time, potatoes are heated faster and the temperature increases faster, which enhances the formation of acrylamide. In addition, the acrylamide content of microwave-fried samples was related to the moisture content [85]. It was obvious that the content of acrylamide increased with a decrease of moisture content. Due to the internal pressure of steam water, the heat transfer is very fast, which can rapidly dry the product without overheating its surfaces, so the moisture content of MVF samples is higher. At a certain humidity level, water has a protective effect on the sample. Keeping the surface of the product moist therefore limits the formation of acrylamide [86]. The flow of water in the vapor state may remove part of the acrylamide and its precursors. Due to volume heating and a higher water flow rate, this volatilization can be intensified when microwave power is used [87]. Therefore, it is necessary to control the water content of the food sample. The drying of a product during cooking leads to a significant increase in the acrylamide content, and so, keeping a product moist throughout cooking can effectively limit the amount of acrylamide formed. 

In brief, MVF can reduce acrylamide formation to a certain extent compared to conventional frying. There are three main factors that may account for this phenomenon: (1) the decrease of frying time; (2) the reduction of frying temperature; and (3) the vapor flow from the center of the sample prevents the formation of acrylamide and its precursors (most important one).

### 3.3. Effect of Microwave Vacuum Frying on Food Quality 

Compared to vacuum frying, microwave-assisted vacuum frying has lower frying temperatures, which leads to a lower oil content, more attractive colors, and better preservation of nutrition and flavor [88]. According to the reports of Su et al. [41], the quality of microwave vacuum-fried potato chips is significantly higher than that of vacuum-fried potato chips. The oil uptake of potato chips was significantly reduced by microwave vacuum frying. The effects of microwave vacuum, vacuum, and conventional frying on food products are shown in Table 4.

The moisture content in foods produced using MVF has been reported to decrease faster than in those produced using vacuum frying, and the higher microwave power levels removed the moisture content from the samples faster [41]. This is because the application of microwaves heats the sample throughout and enhances the outward movement of water, thereby accelerating the heat transfer process, increasing the dehydration rate, and shortening the frying time [83]. In general, the higher the frying temperature and vacuum used, the faster the dehydration is, because the boiling point of water decreases with decreasing pressure.

It has been reported that the application of microwaves during vacuum frying reduced the final oil content of the potato chips produced by about 22–31%, with the oil content decreasing with increasing microwave power [41]. Other researchers reported that the oil content of samples produced using conventional frying was around 12–13%, while that produced using microwave frying was around 9–12% [89]. However, the factors affecting oil absorption during microwave frying are still not fully understood. Oztop et al. [90] hypothesized that the high evaporation rate of water during microwave frying limits the diffusion of oil into the product. However, Moreira et al. [91] hypothesized that the absorption of oil by potatoes mainly occurred during the cooling period, rather than during the frying process, so it is the lower frying temperature required for MWF that reduces the absorption of oil.

In view of the influence of microwaves on the water and oil content of the final products, the texture of microwave fried products is also altered compared to conventional fried products. Quan et al. [79] reported that the application of microwaves during vacuum frying significantly reduced the crushing force, which made the final products harder. This conclusion was consistent with the research of Su et al. [41], who reported that the breaking force (that makes the product crispier) of fried potato chips in the MWF decreased significantly. Because of the application of microwave energy, the evaporation rate of water is faster, which leads to a higher pore density within the potato chips. A more porous structure would be expected to increase the crispness of chips. Su et al. [84] also proposed that the application of microwaves increases the porosity of fried foods, which increases their crispness, thereby improving their textural attributes. 

A microwave treatment has also been shown to significantly improve the brightness (L*) of potato chips. The brightness of a fried food depends on the scattering of light waves by their porous structure, which depends on the number and size of the pores present. The characteristics of the pores in a fried food is influenced by microwave vacuum frying, which therefore impacts its color. For instance, Sahin et al. [85] reported that the lightness of fried potatoes decreased with increasing frying time, while the a* (red-green) and b* (blue-yellow) values did not change significantly. On the whole, microwave frying significantly reduced the overall color change (ΔE*) during cooking [41]. Parikh and Takhar also reported that MVF chips are lighter and yellower than vacuum and conventional fried samples [43].

## 4. Air Frying

Air frying directs hot air containing oil droplets around raw food materials to cook them. The main objective of this kind of frying is to promote the uniform contact between the food and the oil droplets within the hot air stream, which reduces the total amount of oil required to achieve effective cooking. Air frying may therefore lead to final products with greatly reduced fat and calorie contents. 

As a result, this technique not only brings great health benefits, but also has environmental advantages, such as reducing fuel consumption and emissions. However, due to the limitations of the physical form of the oil used to promote heat transfer, some physical properties of the final products may be adversely affected, including texture, color, flavor, and moisture content [92].

### 4.1. The Operating Principles of Air Frying

**Figure 3:**
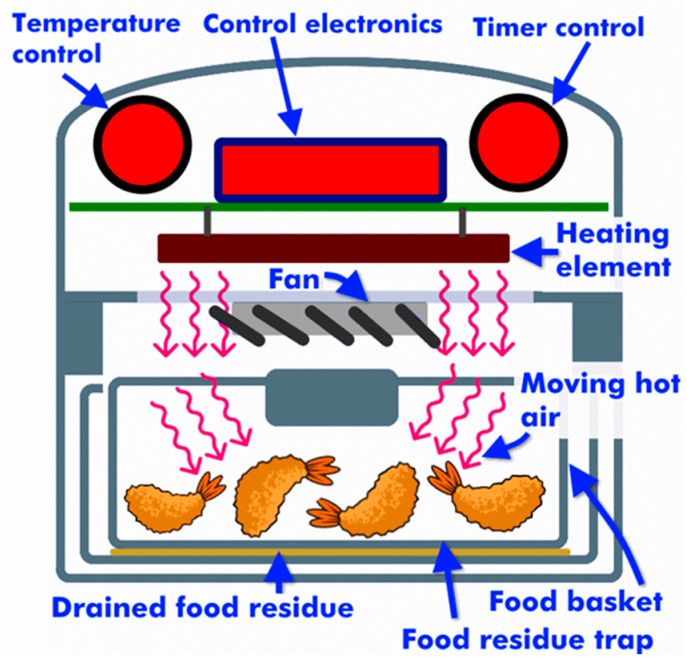
A schematic diagram of air frying equipment, which shows how an air fryer works and the main sections inside [96].

The operating principles of a small-scale air fryer are depicted schematically in Figure 3. An air fryer heats a food product using a dispersion of oil droplets suspended in hot air [93]. This process takes place within an air fryer chamber, which simulates the flow of hot bulk oil in a conventional frying device. Air frying can give similar product characteristics as conventional frying, but oil absorption by the food is usually lower. Indeed, researchers have shown that oil absorption can be reduced by as much as 90% using this method [94]. During air frying, oil droplets are dispersed into a hot air stream by the air fryer so that the surfaces of the food are heated uniformly. The mist containing the oil droplets therefore comes into direct contact with the food inside the air fryer chamber [46]. The product is made to move constantly so that it remains in contact with a fresh super-heated oily mist. During this process, the food heats up, the water is evaporated, and a crust gradually appears on the surface [95]. Because of the high velocity of blowing air and the aid of a circulating fan to move the air inside the chamber, the whole process is considerably shorter than conventional frying.

### 4.2. Effect of Air Frying on Food Safety

Products fried in air tend to be healthier because they contain less fat and potentially harmful substances. According to Basuny et al. [97], the levels of potentially toxic substances in oils extracted from fried foods, such as Maillard and lipid oxidation reaction products, were significantly higher for conventional frying than air frying. 

Acrylamide is a Maillard product formed by the reaction of amino acids (particularly asparagine) and reducing sugars (particularly glucose and fructose) when the temperature of a food exceeds 120 °C during frying or baking. Acrylamide is commonly produced during the frying of foods with high starch contents. Basuny et al. [97] found that the acrylamide content of air-fried potatoes was significantly lower than that of deep-fat-fried potatoes. The acrylamide levels of traditional fried potatoes were 290 ppm, whereas those of the air-fried ones were 78 ppm. In other words, the content of acrylamide in air-fried potatoes decreased by 73.11% compared with that in traditional fried samples. The lower acrylamide level in air-fried potato chips is due to the fact that the frying temperature is lower than the traditional fried temperature. It has been reported that a greater extent of PAH formation occurs as the degree of unsaturation of the added lipids increases [98]. This phenomenon was attributed to unsaturated fatty acids being more prone to oxidation during heating [99]. Therefore, the results indicated that the unsaturated fatty acids present in the frying oil promoted PAH production. The reason why air-fried samples exhibited lower acrylamide and total PAH contents than deep-fat-fried ones can therefore be attributed to the much lower oil content used [100]. 

Basuny et al. [97] reported that the polymer content in foods produced by conventional and air frying were around 0.20 and 0.07%, respectively. Moreover, the concentrations of oxidized fatty acids present after cooking increase to 0.13 and 0.06% for conventional and air frying, respectively. Finally, the free fatty acid content increased from 0.09 to 0.22% in foods produced by conventional frying, while it only increased from 0.09 to 0.12% in foods produced by air frying. The presence of free fatty acids is often used to monitor changes in oil quality during frying [101], with lower levels being more favorable, which again highlights the potential benefits of air drying in improving oil quality.

Feng et al. [102] reported that the reduction in fat content in foods produced by air frying translates into lower postprandial triglyceride (ppTG) responses, which may have health benefits.

### 4.3. Effect of Air Frying on Food Quality 

One of the main drawbacks of air-fried products is a reduction in their sensory attributes, such as a drier mouthfeel and an alteration in their flavor profile, when compared to foods produced by conventional deep-fat frying. The influence of conventional and air frying on the quality attributes of samples are compared in Table 5.

As mentioned earlier, the conventional frying of foods usually involves counteracting mass transport processes: (1) the movement of water from the food into the surrounding hot oil; and (2) the movement of oil from the surrounding hot oil into the food product [103]. The relative rates of these two processes determines the total amount of oil present within the final fried product. 

The decrease in the moisture content of foods has been reported to be slower during air frying than conventional frying [46]. For example, Fang et al. [40] found that the moisture content of tilapia skin decreased from around 69 to 2% in only 6 min for deep-fat frying but in around 10 min for air drying. This difference in the moisture loss rate can be attributed to the fact that the heat transfer rate is much faster in a liquid phase (bulk oil) than in a gas phase (oil mist).

The uptake of oil during air frying has been reported to be up to 90% less than during conventional frying [94]. Carla et al. [93] compared the effects of different vegetable oils (sunflower oil, soybean oil, canola oil, olive oil) used in air frying and traditional frying on the quality of potatoes. The results showed that the fat content of air-fried potatoes decreased by an average of 70%, especially when fried in olive oil. Potatoes have a lower fat content. Teruel et al. [46] compared the effects of conventional and air frying on the quality of French fries and found that the oil content of air fries (0.4 to 1.1 g/100 g sample) was significantly lower than that of traditional fries (5.6 to 13.8 g/100 g sample). Similar results were obtained by Carla et al. [93], who treated potatoes with traditional and air frying. The results showed that fried potatoes produced by air frying had an average oil content of 70% lower than that of conventional fried potatoes. Similarly, Abd et al. [104] found that the fat content of sweet potato samples decreased by 90.1% compared with deep fat-fried samples. Tarmizi and Niranjan [105] studied changes in oil absorption during deep frying, and confirmed that it mainly occurred at the end of the frying process, which was attributed to the condensation of water vapor within the food caused by the decrease in temperature. As a result, some of the oil at the surfaces of the food product were pulled into the voids formed inside. During frying, water and steam are first discharged from the capillaries and then replaced by hot oil. According to Teruel et al. [46], the differences in the final oil content of foods produced by air and conventional frying are due to the “frying medium” surrounding the products: hot oil in the case of deep fat frying, and a mist of oil droplets in air in the case of deep fat frying.

There are also differences in the textural attributes of fried foods produced by air frying as a result of differences in the rate and extent of oil uptake, as well as the kinetics of heat and water transport processes. The frying temperature, frying time, and microstructure of the food crust also play an important role [106]. In terms of appearance, the extent of brownness and evenness of cooking were not significantly different between air-fried and deep fat fried samples, which is also in agreement with instrumental color measurements. However, air-fried samples appeared puffed and dry, whereas deep-fat fried ones gave an oily mouth coating and greasy touch. The “mealiness” sensation reported in air-fried potatoes has been linked to an increase in the volume of gelatinized starch in their cells [107]. The hot-air fried samples also demonstrated greater crust shrinkage during cooling [108]. However, hot-air fried samples retained higher levels of vitamins and had improved oil quality since chemical degradation reactions are inhibited [109].

The effects of hot-air and deep-fat frying on the microstructure, starch gelatinization, and digestibility of potato strips has been investigated [110]. Less starch gelatinization and more starch digestibility were observed in the hot-air fried samples than in the deep-fat fried ones. Hot-air frying produced samples with a compact and well-organized microstructure, while deep-fat frying produced samples with oil-soaked and crusty surfaces.

Color is another important quality attribute of fried foods. Yu et al. [92] reported that the color of surimi products progressively changed with increasing frying temperature and time during hot-air frying, which was attributed to the loss of moisture, a reduction in light reflection, and the formation of brown colors on the surfaces. Other researchers have also reported that the Maillard reaction produces brown crusts on hot-air fried products [111]. According to Abd et al. [104], air-fried sweet potatoes showed lighter color than traditional fried potatoes. In summary, air frying tends to decrease the darkness of the samples in comparison to deep fat frying, especially in frozen samples [109].

## 5. Others

### 5.1. Water–Oil Mixed Frying

Water–oil mixed frying is another innovative frying technology that has advantages over conventional deep-fat frying for certain foods. In this approach, the food residue falls into water, which prevents it from depositing in the oil and being fried repeatedly. The water–oil mixing technology reduces the occurrence of coking and carbonization of residues in oil, which slow-downs the increase in turbidity of frying oils and reduces the deterioration of their quality [113].

In the water–oil mixed deep-frying process, oil and water are added to the same open container, and the oil with relatively low density occupies the upper half of the container, while the water with higher relative density occupies the lower half. An electric heating pipe is placed horizontally in the oil layer of the container. The food is located in the oil layer during frying. A horizontal cooler is installed at the oil–water interface and a forced circulating fan is installed to cool the water, so that the temperature at the oil–water boundary is controlled at 55 °C. The food residue from the fried food falls from the high-temperature oil layer and accumulates in the lower-temperature water layer at the bottom. At the same time, the oil contained in the residue is separated through the water layer and then returned to the oil layer, and the residue falling into the water can be discharged with the water. Ma et al. [35] studied the effect of water–oil mixed frying on soybean oil and chicken. Compared with traditional frying, water–oil mixed frying not only delayed the oxidative deterioration of the soybean oil, but also reduced the production of acrylamide in the chicken.

### 5.2. Spray Frying

In spray frying, a heated oil is sprayed on the sample. Udomkun et al. [33] reported that the moisture content of spray-fried rice crackers was around 18% higher than those of deep-fried samples, while oil uptake was about 32% lower. These researchers also found that the moisture loss and oil uptake increased as the spraying rate was increased. Spray-frying produced rice crackers that had a more attractive color but worse textural properties than those produced by deep frying. This may be one reason why there is currently a lack of information regarding this technique at both research and commercial scales. To improve the spray-frying process, aspects such as frying temperature, frying time, spraying rate, and spinning speed should be optimized.

### 5.3. Radiant Frying

Radiant frying is accomplished by using high-temperature radiant emitters that mimic the heat flux profile which foods typically experience during deep-fat frying. Through adjustment of power settings, emitter-sample geometry, and product exposure time, operators can match the radiant heat flux profile to that of oil immersion frying. Nelson et al. [47] reported that radiant fried chicken patties had 16% less fat and 19% more water than those produced by conventional deep fat frying. However, the crispiness and appearance of the products produced by radiant frying were not satisfying according to a sensory analysis: the color of the radiant fried samples was 11% deeper than the deep-fried ones, and they were not as crispy.

### 5.4. Ultrasonic-Microwave-Assisted Vacuum Frying (UMVF)

**Figure 4:**
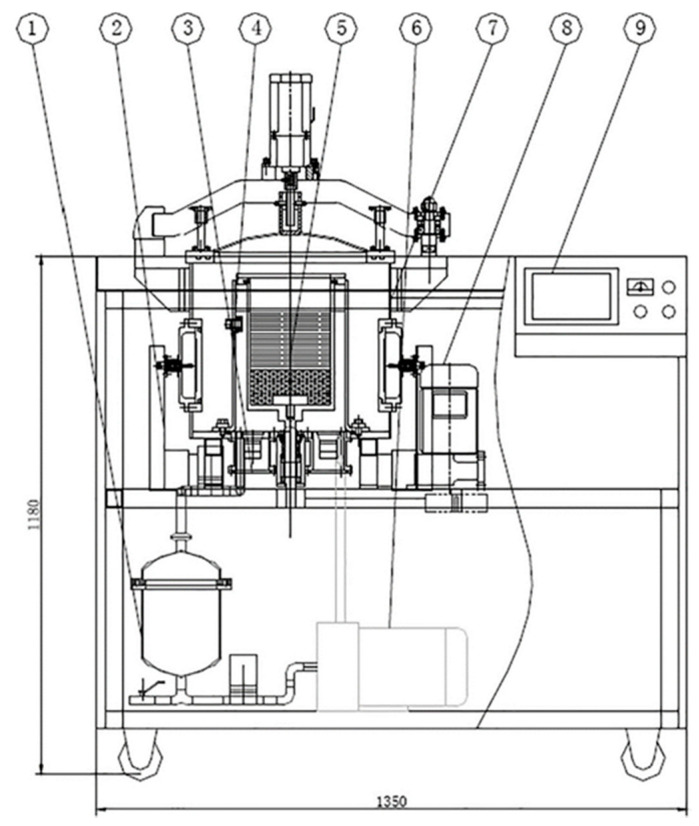
Schematic diagram of the combined ultrasonic-microwave-assisted vacuum frying (USMVF) instrument: (1) Oil tank; (2) microwave source and heating system; (3) ultrasound source and vacuum pressure balance system; (4) vacuum chamber; (5) frying chamber; (6) circulation pump; (7) electric cabin door system; (8) blending and centrifugation system; and (9) controller and operation panel [45].

Ultrasonic-microwave-assisted vacuum frying systems combine an ultrasound source, a microwave source, and a heating system in a vacuum frying system. The microwave source promotes even heating throughout the vacuum chamber, while a series of ultrasonic sources located at the bottom of the frying container are used to ensure thorough mixing. The operating principles of this kind of innovative frying device are shown schematically in Figure 4.

High-energy ultrasonic waves (20–100 kHz) create a series of compressions and rarefactions that can break-up and mix the fluids they are passing through. In addition, cavitation, microstreaming, and channeling effects can promote mass transfer and evaporation processes [114], which can accelerate dehydration processes.

Compared to microwave vacuum frying (MVF), UMVF can significantly improve the water evaporation rate, shorten the frying time, increase the texture quality, and produce a better final color, although the final oil absorption of UMVF is similar to that of MVF, as no reduction of oil content was observed. It has been reported that the combined application of ultrasound and microwave energy had a significant effect on the energy utilization rate and quality index of vacuum-fried potato chips, while shortening frying times by 36 to 55% [45]. In addition, the Maillard reaction products produced by UMVF were less than those produced by MVF. The additional energy provided by the ultrasonic waves decreases the frying temperature required, thereby reducing the formation of harmful substances (such as acrylamide) and improving food quality (color and flavor). From the industrial point of view, UMVF could save energy and extend the shelf life of fried products. Faruq et al. found that the dehydration rate of fruits and vegetables decreases markedly and the entire dehydration process is enhanced during UMVF, giving a more efficient way to produce snacks from fruits and vegetables [82]. UMVF therefore appears to be more effective than microwave vacuum frying. However, it is worth noting that the ultrasonic waves generated by the machine when it is working may pose a threat to the health of the operators and affect their hearing and attention [115]. Therefore, it is necessary to ensure the sound insulation of the instrument, although it will increase the price.

### 5.5. Electric Field Frying (EFF)

Electric field frying (EFF) utilizes high-frequency, low-voltage electric fields to promote the dehydration of foods during frying. EFF does not act directly on the food, but on the frying oil, thereby doing little damage to the food tissue. Studies have shown that the total polar compound content, acid value, viscosity, and color-deepening rate of frying oil increased more slowly when an electric field was applied. The time taken for the total polar compound content of the oil to exceed 27% and the acid value to exceed 5 mg KOH/g, was 32 h for EFF treatment and 28 h for conventional deep frying. The darkening of frying oil reflects the degree of deterioration. Compared with deep-fried oil, the EFF oil had a lighter color and higher transparency at the same frying time, which is indicative of a reduction in the oil deterioration rate during frying. According to Yang et al. [36], fried shrimps prepared using EFF had a higher water content, lower oil content, less microstructure damage, and more uniform oil distribution than those produced by conventional deep-frying. The holes and fractures in the fried shrimp produced by EFF were less, and the final structure was more compact. This kind of structure is not conducive to the absorption and retention of oil, thereby leading to a lower final oil content [116].

## 6. Conclusions and Future Research Directions

In this article, a number of alternative frying methods have been reviewed. Vacuum frying, as one of the earliest and most mature alternative frying methods, still has some problems that are difficult to solve, such as long processing times, the production of “off” flavors, and high equipment costs. However, utilization of a combination of microwave and ultrasonic waves can be used to overcome these problems to some extent. These results suggest that it is feasible to improve the efficacy of alternative frying methods by combining different technologies. As a result, it may be possible to improve the quality and nutritional value of fried starchy products, as well as develop new products. Nevertheless, a lot of research is still needed to clarify the physicochemical mechanisms involved in different innovative frying technologies and to create commercially viable processing operations. The cost of air-frying is relatively low, and it has already entered the kitchen of many ordinary families, but there are still some problems with the taste of the products produced using this method. 

In the future, it will be important to further optimize the different kinds of innovative frying technologies available and to compare their relative advantages and disadvantages for large-scale commercial applications. This may lead to a new generation of high-quality and healthier fried products, which could help reduce the recent increase in many diet-related chronic diseases.

## Figures and Tables

**Table 1:** Fatty acid composition, advantages and disadvantages of different edible oils.

Edible Oil	Concn (g/100 g of Fat)	Advantages	Disadvantages	Ref.
Soybean oil	C18:1, oleic acid: 25.76 ± 0.21 C18:2, linoleic acid: 51.04 ± 1.26 C16:0, palmitic acid: 11.02 ± 0.03	The content of vitamin E is rich; The output of the world is high and the price is cheap.	The high content of polyunsaturated fatty acids makes it easy to oxidize, and regarding rancidity, it smells like fishy beans.	[37]
Rapeseed oil	C18:1, oleic acid: 57.7 ± 0.10 C18:2, linoleic acid: 19.0 ± 0.03 C16:0, palmitic acid: 4.74 ± 0.01	It has high content of monounsaturated fatty acids.	The price is relatively high.	[38]
Sunflower oil	C18:1, oleic acid: 26.1 ± 0.06 C18:2, linoleic acid: 58.5 ± 0.10 C16:0, palmitic acid: 6.44 ± 0.03	It is rich in vitamin E and chlorogenic acid with antioxidant activity.	The content of polyunsaturated fatty acids is high and it is easy to oxidize and deteriorate.	[38]
Peanut oil	C18:1, oleic acid: 41.07 ± 0.43 C18:2, linoleic acid: 40.01 ± 0.62 C16:0, palmitic acid: 11.63 ± 0.03	It has unique peanut flavour and high thermal stability; It can be fried for a short time.	There may be a small number of phospholipids, so frying makes it easy to foam, or even overflow the pan.	[37]
Peanut oil	C18:1, oleic acid: 41.07 ± 0.43 C18:2, linoleic acid: 40.01 ± 0.62 C16:0, palmitic acid: 11.63 ± 0.03	It has unique peanut flavour and high thermal stability; It can be fried for a short time.	There may be a small number of phospholipids, so frying makes it easy to foam, or even overflow the pan.	[37,39]

**Table 2:** Brief on new frying techniques.

Frying Techniques	Characteristics	Applications	Ref.
Vacuum frying (VF)	Low fat content Smooth and uniform microstructure Longer frying time	The efficiency of vacuum frying technology is not high, so microwave and ultrasonic assisted frying can greatly improve the frying efficiency, which is conducive to the expansion of industrial production. However, the equipment is expensive, and the operation and assembly are complex, so it is still difficult to carry out large-scale mass production.	[40]
Microwave vacuum frying (MVF)	Low moisture content Reducing the damage of food nutrients Similar taste of ordinary French fries		[41,42,43]
Ultrasound combined microwave vacuum frying (UMVF)	Increasing evaporation rate compared with VF and MVF Improving texture (crispness) and color, greatly shortening the frying time		[44,45]
Air frying (AF)	Reducing the amount of acrylamide and polar compounds Very low in fat Longer processing time Low degree of starch gelatinization Bad taste compared to traditional fried products	There is no need to add extra oil when frying food, resulting in very low oil content in the final product. This makes it suitable for middle-aged and elderly people with cardiovascular and cerebrovascular diseases, such as hypertension or hyperlipidemia. The price is low and has entered thousands of households. However, its low frying efficiency is not suitable for industrial production.	[32,46]
Oil-water mixed frying (OWF)	Delaying the oxidation and polymerization degradation of oil No obvious change on the water content compared with conventional frying Similar in color, smell and flavor of conventional frying	OWF has less decline in color, smell, flavor, and overall acceptability, which can produce healthier and better fried meat products. It is suggested that measures should be taken to slow down the hydrolysis rate of oil in the process of oil–water mixed frying in the future.	[35]
Spray frying	Low fat content Better color of products (lighter)	In order to improve the spray frying process, various parameters need to be optimized.	[33]
Radiant frying	Lighter in color Less oil and more moisture than the immersion fried products	Sensory evaluation personnel believe that the fried products taste no different from ordinary products, indicating that the fried products can be used as a feasible alternative to oil immersion frying.	[47]
Electric field frying (EFF)	Delaying the oil degradation during frying Lower moisture content Smaller structural damage	This technology can reduce the cost of industrial production, and future work will focus on the mechanism of electric field on fried products to improve the equipment.	[36]

**Table 3:** Effects of vacuum frying on different samples.

Material (Samples)	Treatment	Properties/Effects						Ref.
		Acrylamide (ppd)	Funan (ng/g)	Moisture Content (g/100 g db)	Oil Content (g/100 g db)	Euclidean Distance ΔE	Maximum Breaking Force F (N)	
Matrix constructed by starch	CF (165 °C, 5 min)	-	-	-	-	49.52 ± 3.78	1.80	[72]
	VF (136 °C, 5 min, 33.21 kpa)	-	-	-	-	9.09 ± 0.23	0.70	
Potato chips	VF (100 °C, 10 min, 85 kpa)	-	-	-	0 to 39.14	23.37 ± 0.32	-	[41]
Potato chips	VF (90 °C, 16 min, 10 kpa)	-	-	28.75 to 4	0 to 19.98	-	-	[45]
Potatoes slices	CF (160 °C, 3.8 min)	-	200	-	0 to 49	-	4.20	[65]
	VF (106 °C, 5 min, 10.01 kpa)	-	100	-	0 to 38	-	5.10	

CF, conventional frying; MVF, microwave vacuum frying; db, dry basis.

**Table 4:** Effects of microwave vacuum frying on different samples.

Material (Samples)	Treatment	Properties/Effects						Ref.
		Acrylamide (ug/kg)	Reduction of Acrylamide (%)	Moisture Content (g/100 g db)	Oil Content (g/100 g db)	Euclidean Distance ΔE	Maximum Breaking ForceF (N)	
Potato chips	MVF (100 °C, 10 min, 20 w/g)	-	-	-	0 to 31.92	6.63 ± 0.30	-	[41]
	MVF (120 °C, 10 min, 20 w/g)	-	-	-	0 to 25.13	17.14 ± 7.49	-	
Potato strips	CF (180 °C, 7 min)	138	0	85 to 49	0 to 19	16 ± 5	18	[42]
	MVF (180 °C, 6 min, 430 W)	90	38	85 to 42	0 to 7.3	20 ± 2	59	
	MVF (180 °C, 6 min, 600 W)	46	79	85 to 56	0 to 9	25 ± 3	44	

CF, conventional frying; MVF, microwave vacuum frying; db, dry basis.

**Table 5:** Effects of air frying on different samples.

Material (Samples)	Treatment	Properties/Effects					Ref.
		Acrylamide (ppd)	Polymer Content (%)	Moisture Content (g/100 g db)	Oil Content (g/100 g db)	Euclidean Distance ΔE	
Potato chips	CF (180 °C, 6 min)	288	0.20	77.5 to 3.2	0 to 42	-	[97]
	AF (180 °C, 10 min, 1400 W)	86	0.08	77.5 to 16.6	0 to 0.0024	-	
Sweet potato snack	CF (150 °C, 8 min)	-	-	-	0 to 48.23	-	[104]
	AF (150 °C, 12 min, 1300 W)	-	-	-	0 to 23.78	-	
Potato strips	CF (180 °C, 6 min)	232	0.16	-	-	-	[112]
	AF (180 °C, 6 min, 1300 W)	118	0.10	-	-	-	

CF, conventional frying; AF, air frying; db, dry basis.

## Data Availability

Not applicable.
